# Supplementary material for: Buffering and total calcium levels determine the presence of oscillatory regimes in cardiac cells
Source: PLoS Comput Biol. 2020 Sep 24;16(9):e1007728. doi: 10.1371/journal.pcbi.1007728 (PMC7537911; doi:10.1371/journal.pcbi.1007728)
Supplement: S1 File — In this supplemental information file, we give a full description of the subcellular model, with a table of the different parameters used, as well as the effect of including inactivation in the RyR2. We also show how to obtain the minimal model from a reduction of a compartmental model of a CaRU. (PDF) [file pcbi.1007728.s001.pdf]

Buffering and total calcium levels determine the presence of oscillatory regimes in cardiac cells

Miquel Marchena[1], Blas Echebarria[1] , Yohannes Shiferaw[2] , Enrique Alvarez-Lacalle[1]

1 Departament de Física. Universitat Politècnica de Catalunya-BarcelonaTech, Spain
2 Physics Department. California State University, Northridge. USA

1 Description of the subcellular model

We describe in detail the subcellular calcium cycling model used to investigate the presence of oscillations and the effects of buffering on their characteristic. We have considered the model described in Marchena & Echebarria [1] with the addition of the effect of CSQ and some changes in some of the parameters.

1.1 Spatial structure

The cardiac cell is modeled as a two dimensional domain with $L_x = 100$ μm and $L_y = 15$ μm. We consider a bidomain model, where a fraction of each volume is occupied by the cytoplasm (v_i) and the complementary fraction by the SR (v_{sr}), given that $v_i + v_{sr} = 1$. The spatial grid belongs to the submicron scale and it is defined as $dx = dy = 0.1\mu$m. There are points of the grid with and without RyRs. A typical RyR has a size of 30nm×30nm. The RyRs are transmembrane proteins located at the surface of the SR, so they form a 2D grid. Thus in each of our grid points we locate a maximum of 9 RyRs. RyRs are grouped into clusters named Calcium Release Units (CaRUs). We consider that all the CaRUs have 36 RyRs. In cardiac cells, CaRUs are arranged periodically in the longitudinal and transversal directions, with some -seemingly Gaussian- dispersion [2]. In our model, CaRUs are placed following a Gaussian distribution centered at the z-lines and with a fixed dispersion σ. We take $\sigma = 0.4$ μm as standard value. The average distance between CaRUs is $T_x = 1.6$ μm and $T_y = 0.5$ μm. Experimental data shows that the SR domain coincides with these z-lines [3]. In this sense, we identify the z-lines with periodic narrow strips (0.3 μm width) with a predefined period (T_x). Let be Ω_c the sarcomere domain, that is, the zone between z-lines and let be Ω_{sr} the zone contained in z-lines and all the contour ($\partial\Omega$). Notice that $\Omega_c \cap \Omega_{sr} = \emptyset$. Besides, we consider the presence of Ca^{2+} buffers: troponin (TnC), Calmodulin (CaM) and SR Ca-binding sites. The TnC buffer affects the cytoplasmic concentration of calcium in the Ω_c domain. The other buffers, calmodulin and SR, affect also c_i but in all the cell, $\Omega_c \cup \Omega_{sr}$. We also consider the presence of calsequestrin (CSQ) in the SR. We assume that all the buffers are immobile.

1.2 Calcium concentration evolution

We define c_i, c_{sr} and c_{bi} as the concentration of calcium in the cytoplasm, the SR, and the concentration of calcium bound to buffers. This description assumes that there are effective

diffusion coefficients $D_i = D_i(v_i)$ and $D_{sr} = D_{sr}(v_{sr})$ that, in an average sense, incorporate the effect of that complex geometry. It implies that both diffusion coefficients are functions of the position, $D_i = D_i(\mathbf{r})$ and $D_{sr} = D_{sr}(\mathbf{r})$. In our simulations we take the values $D_i \sim 250\ \mu\text{m}^2/\text{s}$ and $D_{sr} \sim 90\ \mu\text{m}^2/\text{s}$, that are within the upper range considered in the literature [4, 5].

We state the problem with the following set of partial differential equations (PDEs).

$$\frac{dc_i(\mathbf{r},t)}{dt} = J_i(\mathbf{r},t) + \nabla \cdot [D_i(\mathbf{r})\nabla c_i(\mathbf{r},t)] - J_{bi}(\mathbf{r},t) \tag{1}$$

$$\frac{dc_{sr}^{tot}(\mathbf{r},t)}{dt} = \frac{v_i(\mathbf{r})}{v_{sr}(\mathbf{r})} J_{sr}(\mathbf{r},t) + \nabla \cdot [D_{sr}(\mathbf{r})\nabla c_{sr}(\mathbf{r},t)], \tag{2}$$

$$\frac{dc_{bi}(\mathbf{r},t)}{dt} = J_{bi}(\mathbf{r},t), \tag{3}$$

where the dynamics of CSQ has been considered to be fast based on their rate constants [6, 7] compared with the release time scale [8]. For that reason, we have applied the rapid buffer approximation to CSQ. J_i and J_{sr} are the fluxes into the cytosol and the SR spaces, respectively, J_{bi} accounts for the binding of free calcium to the different buffers. The fluxes that may contribute to the total flux into the cytosol J_i are the SR release flux, J_{rel} and the SERCA pump, J_{up}. The model presented in [1] has been modified in order to exclude the effects of L-type Calcium Channels and Na-Ca exchanger, since we are studying the appearance of spontaneous calcium waves.

1.3 Fluxes description

The flux J_{rel} exists only on those points that have a CaRU. J_{up} pumps calcium from the cytoplasm to the SR and it is present in all cell domain ($\Omega_c \cup \Omega_{sr}$). The expression of currents J_i and J_{sr} used in the model description have to be split up in cases. In particular, for the already defined regions (Ω_c, Ω_{sr} and $\delta\Omega$) we have that:

- Inter z-planes space ($\mathbf{r} \in \Omega_c$):

$$J_i(\mathbf{r},t) = -J_{sr}(\mathbf{r},t) = -J_{up}(\mathbf{r},t) \tag{4}$$

  These currents are defined in internal points without RyR.

- Internal cytosolic space along the z-lines ($\mathbf{r} \in \Omega_{sr} \setminus \delta\Omega$):
  - without RyR:

$$J_i(\mathbf{r},t) = -J_{sr}(\mathbf{r},t) = -J_{up}(\mathbf{r},t) \tag{5}$$

  - with RyR:

$$J_i(\mathbf{r},t) = -J_{sr}(\mathbf{r},t) = J_{rel}(\mathbf{r},t) - J_{up}(\mathbf{r},t) \tag{6}$$

In the following section we will give a broad description of the currents used above.

1.4 Electrophysiological currents

Release current. The release current depends on the number of RyR in the open state O according the equation:

$$J_{rel} = g_{rel} O (c_{sr} - c_i) \tag{7}$$

The Markov description of the RyR model with the different states considered is shown in Fig. 1. Transition dynamics between each state (in each cluster) is solved stochastically using a

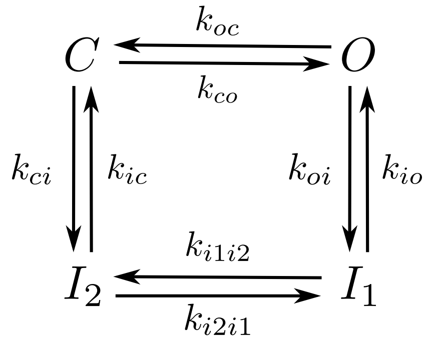

Figure 1: Markov model of the RyR.

time-adaptive Gillespie's method [9]. Cooperativity in activating Ca^{2+} binding is incorporated by transition rates depending on c_i^2. In particular, four transition rates are calcium dependent:

$$k_{co}(c_i) = k_a c_i^2 \qquad k_{i2i1}(c_i) = k_b c_i^2 \qquad k_{oi} = k_c c_i \qquad k_{ci} = k_c c_i \tag{8}$$

The other rates are constant with the following properties $k_{oc} = k_{i1i2}$, $k_{oi} = k_{ci}$. Detailed balance implies $k_{io} = k_{ic}(k_a/k_b)$.

SERCA current. The associated current to the SERCA pump is considered to be:

$$J_{up} = g_{up}\frac{(c_i/K_i)^2 - (c_{sr}/K_{sr})^2}{1 + (c_i/K_i)^2 + (c_{sr}/K_{sr})^2}. \tag{9}$$

J_{up} can be outward or inward depending on the relative concentrations. We say that J_{up} is an inward flux when it increases the concentration in the SR. Otherwise, it will be outward. Since the denominator is always positive, an inward flux is achieved when:

$$\left(\frac{c_i}{K_i}\right)^2 - \left(\frac{c_{sr}}{K_{sr}}\right)^2 > 0 \rightarrow \frac{c_i}{K_i} > \frac{c_{sr}}{K_{sr}}, \tag{10}$$

where K_i and K_{sr} represent the local equilibrium concentration of cytoplasmic and SR, respectively. Since we are considering an homogenized model, the SERCA pump is set in the whole cell.

Buffer dynamics. The dynamics for each of the three buffer concentrations ($c_{b,j}$) in the cytosol is given by

$$J_{b,j} = k_{on,j} c_i (B_T - c_{b,j}) - k_{off,j} c_{b,j} \tag{11}$$

with j representing TnC, CaM or SR buffers. On the other hand, CSQ is approximated with the Rapid Buffer Approximation. Let be q the amount of free CSQ, that is, $q = B_{CSQ} - c_{b,CSQ}$. The reaction that governs this process is

$$\mathrm{c}_{sr} + \mathrm{q} \underset{\mathrm{k_{off,CSQ}}}{\overset{\mathrm{k_{on,CSQ}}}{\rightleftharpoons}} \mathrm{c_{b,CSQ}}$$

and the ODE that describes this reaction is

$$\frac{d[c_{b,CSQ}]}{dt} = -k_{off,CSQ}[c_{b,CSQ}] + k_{on,CSQ}[c_{sr}][q] \tag{12}$$

Since we assume that the CSQ buffering binding process is faster than release, Eq. (12) can be taken to be in steady state. Then,

$$0 = -k_{off,CSQ}[c_{b,CSQ}] + k_{on,CSQ}[c_{sr}][q] \tag{13}$$

The total concentration of CSQ in the SR is

$$B_{CSQ} = q + c_{b,CSQ} \tag{14}$$

Combining both equations, one can obtain an equation for the free concentration of CSQ

$$q = \frac{B_{CSQ}K_{CSQ}}{K_{CSQ} + c_{sr}} \tag{15}$$

where K_{CSQ} is the dissociation constant and it is defined as $K_{CSQ} \equiv k_{off,CSQ}/k_{on,CSQ}$. The concentration of buffer bound to CSQ is

$$c_{b,CSQ} = B_{CSQ} - q = B_{CSQ}\left(1 - \frac{K_{CSQ}}{K_{CSQ} + c_{sr}}\right) = \frac{B_{CSQ}c_{sr}}{K_{CSQ} + c_{sr}} \tag{16}$$

The total concentration of calcium in the SR is the sum of the free calcium and the calcium bound to CSQ

$$c_{sr}^{tot} = c_{sr} + c_{b,CSQ} = c_{sr}\left(1 + \frac{B_{CSQ}}{K_{CSQ} + c_{sr}}\right) \tag{17}$$

and the inverse relation is

$$c_{sr} = \frac{1}{2}\left[c_{sr}^{tot} - K_{CSQ} - B_{CSQ} + \sqrt{(c_{sr}^{tot} - K_{CSQ} - B_{CSQ})^2 + 4c_{sr}^{tot}K_{CSQ}}\right] \tag{18}$$

In the simulation, we solve the PDE for c_{sr}^{tot} and then we calculate the free calcium in the SR applying the Eq. (18). Computationally, with this procedure the numerical error of the simulations in the RBA of CSQ is negligible.

The parameters used in the subcellular model are detailed in Table 1. They are split in general structural parameters of the model, buffer parameters and key physiological values of SERCA and RyR.

2 SR luminal dependence

For simplicity, and for a better comparison with the results of the minimal model (cf. Eqs. (18)-(19) in the main manuscript), in the results of the subcellular model shown in Figs. 3, 4 of the main manuscript we have not considered the dependence of the SR luminal calcium on the probability of transition to the open state of the RyR. To include this dependence we have defined the new open probability $k_{cosr} = k_{co}/f(c_{sr})$, with the shut down function $f(c_{sr})$ following [10]:

$$f(c_{sr}) = \frac{1 + \alpha\left(\frac{EC_{50}}{c_{sr}}\right)^H}{1 + \left(\frac{EC_{50}}{c_{sr}}\right)^H} \tag{19}$$

where EC_{50} is the SR luminal threshold, below which the RyRs open probability is decreased by a factor α, and H is the exponent that defines the steepness of that switch. As in [10], this function $f(c_{sr})$ also affects the inactivation rates. Increasing the load (see Fig. 2), the system

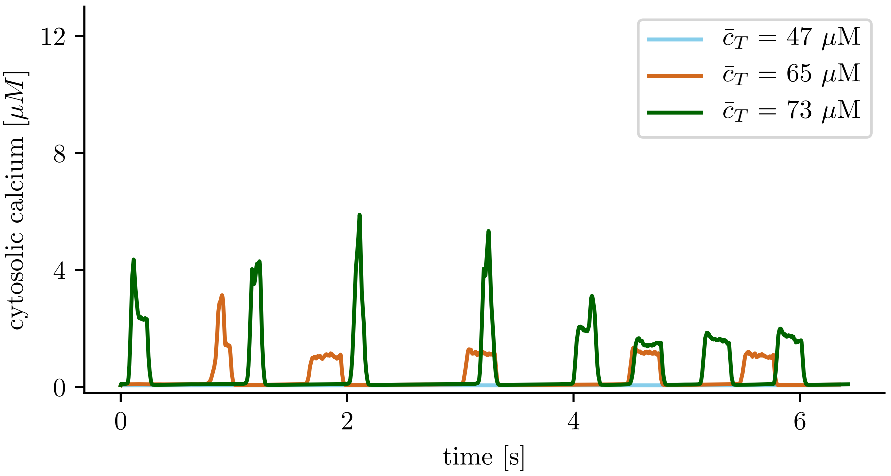

Figure 2: Calcium traces obtained with the full subcellular model and three different values of the average calcium concentration, c_T. The open rate between the open and the closed state has been modified to include a dependence on the luminal calcium following Eq. 19, with $H = 10$, $EC_{50} = 500\mu$M, and $\alpha = 15$.

undergoes a transition from a low cytosolic calcium state, where RyRs remain in the close state, to spontaneous oscillations, giving rise to calcium waves. This transition happens at the a similar value of $\bar{c}_T$ as in the case without SR luminal dependence. However, the transition to the high cytosolic calcium state, where the RyRs remain open, occurs at larger values of the total calcium content. Thus, these simulations suggest that the range of oscillations is broader when the SR dependence is included to the model.

3 Full calcium model of a CaRU

In this appendix, we show how to derive the simplified model in Eqs. (12)-(14) of the main paper from a detailed deterministic model of calcium handling. We consider cytosolic and luminal spaces, each separated into different compartments, i.e, dyadic and cytosolic by the one side, and junctional and network SR, by the other. We also consider the effect of two buffers in the cytosol, TnC and SR, and Calsequestrin (CSQ) in the SR. The effect of the buffers is actually very relevant, as they change the structure of possible solutions of the system.

With this, the dynamics can be described by the following set of deterministic equations for the calcium concentration at the different compartments

$$\frac{dc_d}{dt} = gP_o(c_j - c_d) - \frac{c_d - c_i}{\tau_i} \tag{20}$$

$$\frac{dc_{sr}}{dt} = \frac{v_i}{v_{sr}} g_{up} \frac{c_i^2}{K_s^2 + c_i^2} - \frac{c_{sr} - c_j}{\tau_{sr}} \tag{21}$$

$$\frac{dc_j}{dt} = \frac{v_{sr}}{v_{jsr}} \frac{c_{sr} - c_j}{\tau_{sr}} - \frac{v_d}{v_{jsr}} gP_o(c_j - c_d) - k_{onSQ}\, c_j(B_{SQ} - C_{bSQ}) + k_{offSQ} C_{bSQ} \tag{22}$$

where c_d, c_j, c_{sr}, c_i stand for the concentration in dyadic space, free luminal, SR network and cytosol, P_o is the fraction of RyRs in the open state, τ_i and τ_{sr} are the diffusion time constants

out of the dyadic space and SR network, v_i, v_d, v_{jsr} and v_{sr} are the volumes associated with each compartment, g_{up} is the strength of SERCA pump, K_s is the concentration at which SERCA closes and g is the strength of the release current. The dynamics of the buffers are given by linear reactions with the following set of ODEs

$$\frac{dC_{bTnC}}{dt} = k_{onTnC}c_i(B_{TnC} - C_{bTnC}) - k_{offnTnC}C_{bTnC} \tag{23}$$

$$\frac{dC_{bSR}}{dt} = k_{onSR}c_i(B_{SR} - C_{bSR}) - k_{offSR}C_{bSR} \tag{24}$$

$$\frac{dC_{bSQ}}{dt} = k_{onSQ}c_j(B_{SQ} - C_{bSQ}) - k_{offSQ}C_{bSQ} \tag{25}$$

Since the total amount of calcium has just a variation of about by a 5% or 10% over a calcium cycle, we assume that the total calcium concentration, $\bar{c}_T$, is fixed. In this way, one does not have to solve a differential equation for the calcium concentration in the cytosol. Rather, it is derived from the algebraic equation:

$$\bar{c}_T = \frac{v_i}{v_i + v_{sr}}(c_i + c_{b,TnC} + c_{b,SR} + c_{b,CaM}) + \frac{v_{sr}}{v_i + v_{sr}}(c_{sr} + c_{bSQ}) \tag{26}$$

Homeostatic behavior of the cell will eventually load the system more or less, increasing or decreasing $\bar{c}_T$.

Gating of the RyR can been described by Markov models that describe the transitions among different conformations of the channel. Thus, for the dynamics of the RyR we consider a phenomenological four state model [11].

$$\frac{dP_R}{dt} = -k_p c_d^2 P_R - k_i c_d P_R + k_r P_{I_A} + k_m P_o \tag{27}$$

$$\frac{dP_o}{dt} = k_p c_d^2 P_R - k_i c_d P_o + k_r P_{I_B} - k_m P_o \tag{28}$$

$$\frac{dP_{I_A}}{dt} = -k_p c_d^2 P_{I_A} + k_i c_d P_R - k_r P_{I_A} + k_m P_{I_B} \tag{29}$$

with the last equation given by the condition that the sum of probabilities is equal to 1

$$P_{I_B} = 1 - P_R - P_o - P_{I_A} \tag{30}$$

P_R and P_o are the ratios of local RyR in the recovered and open states. P_{I_A} and P_{I_B} stand for the terminated states.

To make the model treatable we assume several hypotheses.

1. We consider rapid equilibrium of c_j ($\dot{c}_j = 0$). Thus

$$\dot{c}_j = 0 = \frac{v_{sr}}{v_{jsr}}\frac{c_{sr} - c_j}{\tau_{sr}} - \frac{v_d}{v_{jsr}}gP_o(c_j - c_d) - k_{onSQ}\, c_j(B_{SQ} - C_{bSQ}) + k_{offSQ}C_{bSQ} \tag{31}$$

then

$$c_j = c_{sr} - \tau_{sr}\left[\frac{v_d}{v_{sr}}gP_o(c_j - c_d) - \frac{v_{jsr}}{v_{sr}}\left(k_{onSQ}c_j(B_{SQ} - C_{bSQ}) - k_{offSQ}C_{bSQ}\right)\right] \tag{32}$$

2. At first order, we approximate c_j as c_{sr} in the right hand side of Eq. (32). This approximation is valid when $\tau_{sr}\frac{v_d}{v_{sr}}gP_o \ll 1$ and $\tau_{sr}\frac{v_d}{v_{sr}}gP_o \ll 1$. Then, the ODE for c_{sr} in Eq.

(22) reads as

$$\frac{dc_{sr}}{dt} = \frac{v_i}{v_{sr}} g_{up} \frac{c_i^2}{K_s^2 + c_i^2} - \frac{v_d}{v_{jsr}} g P_o (c_{sr} - c_d) - \frac{v_{jsr}}{v_{sr}} \left[k_{onSQ} c_{sr} (B_{SQ} - C_{bSQ}) - k_{offSQ} C_{bSQ}\right] \tag{33}$$

where we have eliminated the dependence with c_j.

3. We apply the rapid buffer approximation in the SR because CSQ is very fast [6, 7]. The derivation of c_{sr} in terms of c_{sr}^{tot} have been already shown in Eqs. (10)-(7) of the main manuscript.

4. We assume that all the buffers in the cytosol are in equilibrium. Then

$$C_{bTnC} = \frac{B_{TnC} c_i}{K_{TnC} + c_i}, \quad C_{bSR} = \frac{B_{SR} c_i}{K_{SR} + c_i} \tag{34}$$

5. Besides, we combine both TnC and SR buffers in only one buffer with a total concentration of B_b and an affinity of K_b, which will be

$$C_b = \frac{B_b c_i}{K_b + c_i} \tag{35}$$

6. Finally, since in our simulations the inactivated states of the RyRs are not determinant to produce oscillations (see Fig. S1), for simplicity we have neglected them. For that reason, we reduce the four state model to a two state model

$$\mathrm{C} \underset{\mathrm{k}_m}{\overset{\mathrm{k}_p \mathrm{c}_d{}^2}{\rightleftharpoons}} \mathrm{O}$$

where k_m sets the mean open time $\tau_{rec} = 1/k_m$ of a RyR while k_p gives the open probability. Then, the ODE for the open probability P_o is

$$\frac{dP_o}{dt} = -k_m P_o + k_p c_d^2 (1 - P_o) \tag{36}$$

The parameters of these equations are taken from the literature where, except for those of the RyR, are well documented. The ratio of SR to cytosol in the cell is roughly 1 to 20-50. The order of magnitude of the volume of the cleft where calcium is released is around 10^{-3} μm^3 [10]. SERCA is roughly activated at around 0.2-0.5 μM and closed at around 15-20 μM with the number of buffers relevant to absorption around 50-100 μM, taking an average affinity of around 0.5 μM [10]. The whole set of parameters is given in Table 2.

References

[1] Marchena M, Echebarria B. Computational model of calcium signaling in cardiac atrial cells at the submicron scale. Frontiers in physiology. 2018;9:1760.

[2] Chen-Izu Y, McCulle SL, Ward CW, Soeller C, Allen BM, Rabang C, et al. Three-dimensional distribution of ryanodine receptor clusters in cardiac myocytes. Biophysical journal. 2006;91(1):1–13.

[3] Soeller C, Crossman D, Gilbert R, Cannell MB. Analysis of ryanodine receptor clusters in rat and human cardiac myocytes. Proceedings of the National Academy of Sciences. 2007;104(38):14958–14963.

[4] Louch WE, Hake J, Jølle GF, Mørk HK, Sjaastad I, Lines GT, et al. Control of Ca2+ release by action potential configuration in normal and failing murine cardiomyocytes. Biophysical journal. 2010;99(5):1377–1386.

[5] Bers DM, Shannon TR. Calcium movements inside the sarcoplasmic reticulum of cardiac myocytes. Journal of molecular and cellular cardiology. 2013;58:59–66.

[6] Robertson S, Johnson JD, Potter J. The time-course of Ca2+ exchange with calmodulin, troponin, parvalbumin, and myosin in response to transient increases in Ca2+. Biophysical journal. 1981;34(3):559–569.

[7] Cannell M, Allen D. Model of calcium movements during activation in the sarcomere of frog skeletal muscle. Biophysical Journal. 1984;45(5):913–925.

[8] Donoso P, Prieto H, Hidalgo C. Luminal calcium regulates calcium release in triads isolated from frog and rabbit skeletal muscle. Biophysical journal. 1995;68(2):507–515.

[9] Nivala J, Knowles P, Dotro G, García J, Wallace S. Clogging in subsurface-flow treatment wetlands: measurement, modeling and management. Water research. 2012;46(6):1625–1640.

[10] Shannon TR, Wang F, Puglisi J, Weber C, Bers DM. A mathematical treatment of integrated Ca dynamics within the ventricular myocyte. Biophysical journal. 2004;87(5):3351–3371.

[11] Stern MD, Song LS, Cheng H, Sham JS, Yang HT, Boheler KR, et al. Local control models of cardiac excitation–contraction coupling. The Journal of general physiology. 1999;113(3):469–489.

Table 1: Parameters of the subcellular model.

**General parameters**		
time step	Δt (ms)	
spatial step	Δx (μm)	0.1
Resting potential	V_{res} (mV)	-85
Maximum potential	V_{max} (mV)	10
cell x-length	L_x (μm)	100
cell y-length	L_y (μm)	15
volume factor (cytoplasm)	v_i/v_{sr}	200
volume factor (SR)	v_i/v_{sr}	20
volume myocyte	v_{myo} (μm^3)	21500
Temperature	T(K)	308
Faraday constant	F(C/mol)	96485
Ideal gas constant	R(J/(Kmol))	8.31
**Buffer Parameters**		
TnC concentration	B_{TnC}(μM)	50
TnC binding rate	$k_{on,TnC}$(μM^{-1}ms^{-1})	0.00327
TnC unbinding rate	$k_{off,TnC}$(μM^{-1})	0.00196
Calmodulin concentration	B_{CaM}(μM)	15
Calmodulin binding rate	$k_{on,CaM}$(μM^{-1}ms^{-1})	0.003
Calmodulin unbinding rate	$k_{off,CaM}$(μM^{-1})	0.02
SR-bound buffer concentration	B_{SR}(μM)	15
SR-bound buffer binding rate	$k_{on,SR}$(μM^{-1}ms^{-1})	0.01
SR-bound buffer unbinding rate	$k_{off,SR}$(μM^{-1})	0.006
Calsequestrin concentration	B_{CSQ}(mM)	5
Calsequestrin dissociation constant	K_{CSQ}(mM)	2
**SERCA parameters**		
Maximum uptake SERCA	g_{up}(μMms^{-1})	0.5
Half occupation of cytosolic calcium binding states	K_i(μM)	0.0615
Half occupation of SR calcium binding states	K_{sr}(mM)	1.3
**RyR parameters**		
Single channel strength	g_{rel}(ms^{-1})	4
Number of RyR per CaRU	N_{RyR}	36
Closing rate	$k_{oc} = k_{i1i2}$(ms^{-1})	0.08
Inactivation rate parameter	k_c(μM^{-1}ms^{-1})	0.001
Recovery rate from $I \to C$	k_{ic}(ms^{-1})	0.0025
Opening rate parameter	k_a(μM^{-2}ms^{-1})	0.0014
Transition in I states	k_b(μM^{-2}ms^{-1})	10^{-4}

Table 2: Parameters of the compartmental model, corresponding to a CaRU of size $0.7 \times 0.7 \times 1.4 \mu\text{m}^3$.

Parameters		
Parameter	Units	Value
v_d	μm^3	0.001
v_i	μm^3	0.45
v_{sr}	μm^3	0.01
τ_i	ms	0.01
g_{up}	μM/ms	0.5
K_s	μM	0.2
g	ms^{-1}	20
k_p	μM^{-2} ms^{-1}	10^{-3}
k_m	ms^{-1}	0.25
K_o	μM	15
B_b	μM	80
K_b	μM	0.5
