# Supplementary figures and images for: Buffering and total calcium levels determine the presence of oscillatory regimes in cardiac cells
Source: PLoS Comput Biol. 2020 Sep 24;16(9):e1007728. doi: 10.1371/journal.pcbi.1007728 (PMC7537911; doi:10.1371/journal.pcbi.1007728)

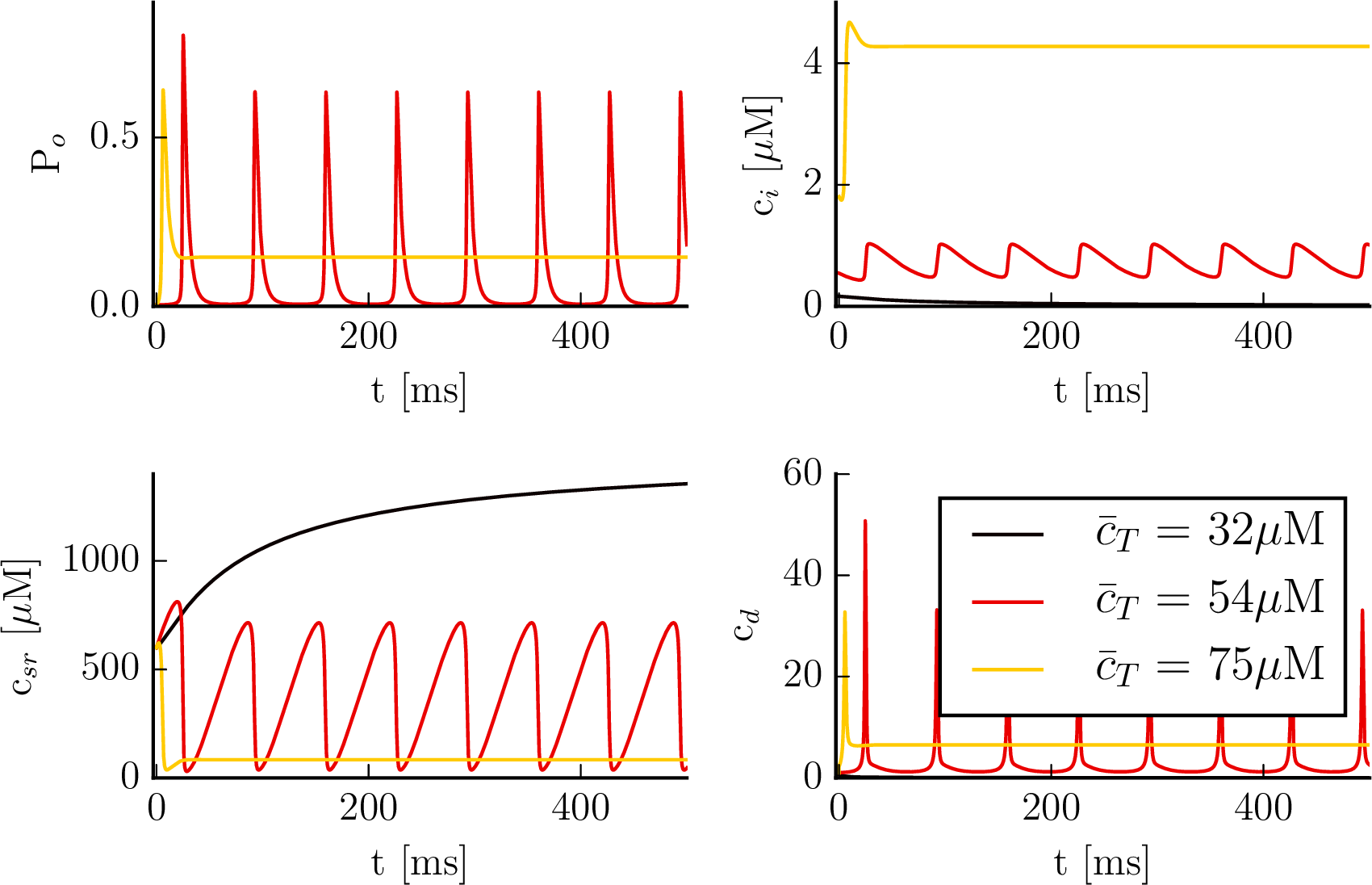

Supplement: S1 Fig — After a transient, the system ends up in either a steady state which is excitatory at low levels of total calcium in the cell with observed low levels of calcium in the cytosol, in an oscillatory state with intermediate levels of total calcium in the cell, or in a state of high total levels of calcium in the cell with observed high cytosolic calcium levels. (TIF) [file pcbi.1007728.s002.tif]

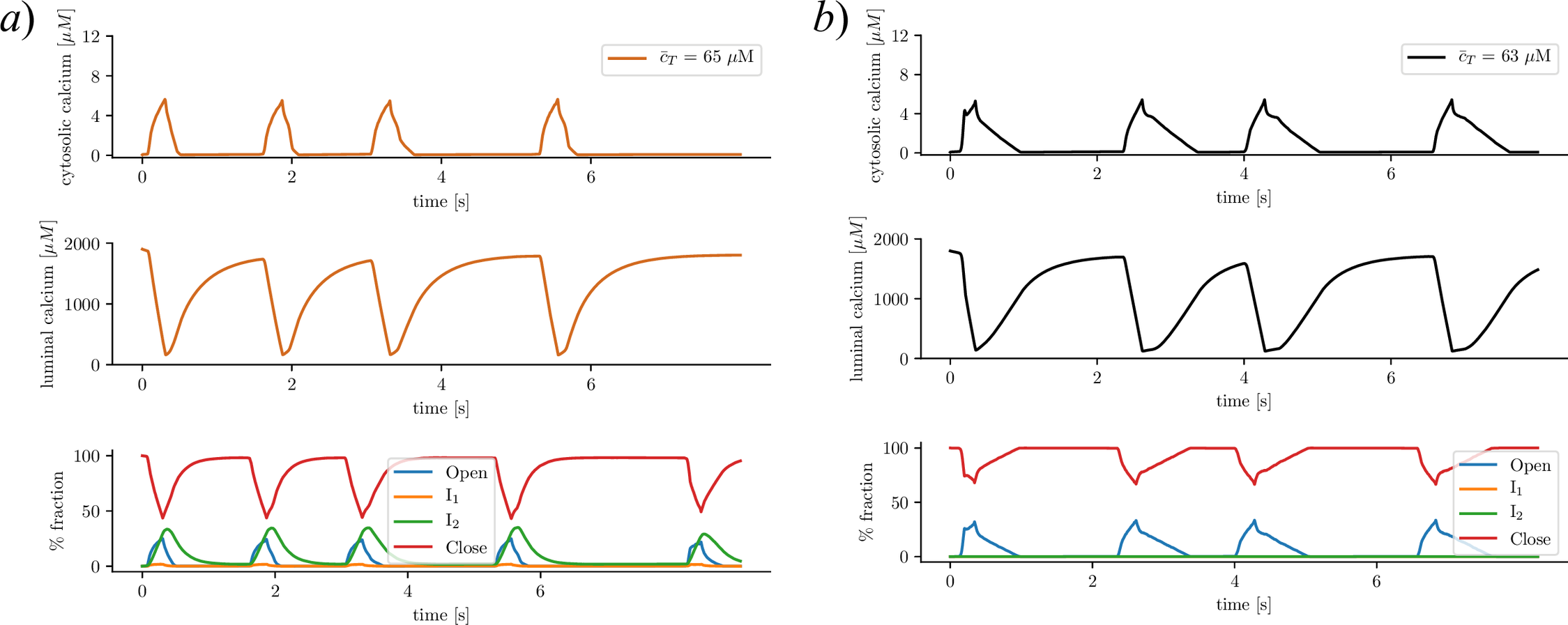

Supplement: S2 Fig — In the lower panel we show the fraction of RyRs in the different states shown in the schematics of Fig 1b. For the simulations in (b) we have set all the inactivation rates equal to zero. (TIF) [file pcbi.1007728.s003.tif]
