# Supplementary material for: Buffering and total calcium levels determine the presence of oscillatory regimes in cardiac cells
Source: PLoS Comput Biol. 2020 Sep 24;16(9):e1007728. doi: 10.1371/journal.pcbi.1007728 (PMC7537911; doi:10.1371/journal.pcbi.1007728)
Supplement: Attachment — Submitted filename: ResponseToReviewers.pdf [file pcbi.1007728.s004.pdf]

Response to reviewer 1:

The manuscript by Marchena et al. explores mechanisms underlying calcium oscillations in cardiac cells using mathematical models. They use a detailed model and a minimal model and show how buffering and total calcium levels affect calcium oscillations. The manuscript is well written and the findings are interesting. I have a few questions and suggestions.

We are glad that the reviewer finds our results interesting. In the new version of the manuscript we have introduced changes (shown in blue) to address all his/her concerns, that we detail below.

*In the minimal model, Po only depends on cd (dyadic calcium). Doesn't luminal Ca regulation of RyRs play a role in oscillations?

We thank the referee for raising this important point. We think that it relates two important issues which link the methodology with the results of the paper and that should have been addressed in more detail in the text.

Luminal calcium regulation of the RyR may certainly play an important role in maintaining calcium homeostasis, and some dysregulations can give rise to spontaneous calcium release leading to DADs or EADs. However, in this paper we were interested in studying the effects of buffers (and, more specifically, CSQ) and global calcium content in the appearance of oscillations, since we think that these are the two main contributors to this instability. This is explained now in the Methods section.

While the aim of the paper was not to investigate the effects of luminal calcium regulation, we think that, for completeness, simulations and analysis regarding this point were needed. Now we include simulations that take into account RyR luminal regulation (in Appendix B), showing that it does not affect the onset of alternans and, if anything, it increasing the region where oscillations are observed. In other words, we show that it can also play a role in the sustaining of oscillations.

We think it is very important to clarify this point to the reader. We have introduced the following paragraph in the methods section with this aim:

We have considered two types of RyR regulation. First, we have considered an open rate and termination dependent on both cytosolic and luminal calcium. The dependence on luminal calcium is due to the presence of CSQ, which mediates its opening, as described in the introduction. However, the core of the paper uses a RyR that is not affected by luminal calcium. One of the key analysis of this paper is to test which are the effects of buffering on the appearance of oscillations purely in terms of its binding properties to calcium and not its regulatory effects. Introducing a CSQ-mediated RyR opening would mix different possible mechanisms. For comparison and analysis, we think it is better to leave this dependence initially out. Once the mechanism of CSQ as a buffer is understood we have used Appendix A to show that a full detailed model of RyR with its open rate dependent on SR calcium content gives exactly the same transition to oscillations as described in the main text. If anything, this regulatory mechanism just makes them more robust.

*Some buffers are fast, and others are slow. How do affinities, kinetics, and concentrations of the different buffers affect your results. Does mobility affect your results?

In this paper we wanted to focus on the effects of changes in the amount of CSQ and in the global cell calcium content, since there are experiments that relate this to the appearance of calcium waves and oscillations. A full study of the effects of the different buffers' characteristics (affinities, kinetics, mobility) is very interesting, but we defer it for further work. The main reason behind not addressing this issue in this manuscript is that a reduction in total buffer concentration can always be reasonably associated with dramatic changes in kinetics and affinity, but mobility cannot. A very high affinity is, in a certain sense, similar to eliminating the presence of buffer but a high mobility cannot be similarly related to it. In other words, a full analysis of this issue requires addressing mobility and its importance. And here, we encounter the problem that the reduced model, by its own nature, does not include buffer mobility. Since we consider the insights of the minimal model key to understand this process, the effects of mobility must be addressed having stablished first the scenario we unveil in this manuscript.

While we do not expect that the main results of this manuscript will be affected under reasonable slow buffer mobility, we agree that investigating the robustness of our results under different mobility scenarios (fast movement of buffers in the cytosol and in membranes) must be addressed in future work.

We address this issue now in the discussion with the following paragraph:

Although in this paper we have focused on the effect of CSQ, an interesting extension of this work would be to study in more detail the effect of changes in cytosolic buffer dynamics, allowing, for instance, for mobile buffers. This would require to modify the minimal model to account for diffusion of the buffer to nearby calcium release units, and we defer it for future work.

*Pg 3: “distributed over the cell along the z-lines with a Gaussian distribution in both transversal and longitudinal axes.” Can you elaborate this sentence? What are the mean and the standard deviation? Is there any experimental evidence?

In order to specify the position of the RyR clusters we define first a rectangular grid, with a distance of 1.6μm in the longitudinal direction and 0.5μm in the transversal direction. Then, we place each RyR cluster around each point of the grid, with a given variance in the transversal and longitudinal directions (σ=0.4μm). There is indeed experimental evidence of this. See, for instance, Fig. 2 in Chen-Izu et al, Biophys J, 2006. We have added these details in the text to clarify this point.

*Pg7: “Waves are normally initiated at different sites each time” This implies that the cell size is also important (i.e. larger cells have more waves). On the other hand, the minimal model is a model of a single CaRU. Can you justify the use of the minimal model to analyze the full subcellular model?

*Fig 5: The oscillation period is too short for cardiac cells.

These two points are strongly related so we address them together. A proper explanation of the second point clarifies the relevance of the minimal model to understand the full subcellular model and the effects of cell size. Further explanation of these points and a more thoroughly discussion in the paper is included now in the manuscript.

The reason behind short oscillations periods in the CaRU is related with noise effects. The deterministic equations for the CaRU provide the proper average behavior of the units and the core behavior of the whole system. However, the period of each unit can be affected close to the transition by small amounts of noise. Due to the fact that there are a small number of RyRs per cluster, the study of periods must include an analysis of noise in the system. It provides a better representation of what occurs in the subcellular model (and in experiments). Close to the onset of a homoclinic bifurcation giving rise to a periodic orbit, a small amount of noise makes oscillations appear for values of the parameters before the onset in the deterministic limit, and with a larger period of oscillation. In fact, in the new Fig. 16 it can be seen that, at onset of oscillations, periods of the order of the second are observed. In the new version of the manuscript we have added a new subsection on the stochastic effects and modified a paragraph on the Discussion to address these results.

Introducing noise in the CaRU provides the proper oscillation period but the analysis of the deterministic minimal model is still useful to analyze the full cell since it determines the minimum period of oscillation and the possibility of having these waves in the first place. If each of the units does not have an oscillatory nature or is very close to having one, the presence of periodic waves is not possible. A single unit can indeed release calcium stochastically but it then produces only sparks. Waves are the coordinating mechanism of the oscillatory substrate of each unit. All units will have a tendency to fire periodically, but noise leads to different periods making some units have a slightly early release. Diffusion of calcium to neighbors triggers the release of other units that were already close to open again.

In a very large cell we would expect to observe more than one initiation site for the waves. However, these different initiation sites would produce waves at approximately the same time fixed by the dynamics of the CaRU. Here we must notice the important nature of the deterministic approach to shed light on this issue. Each CaRU is stochastic but on average has the same behavior. This means that some units will open at slightly different times so the period of the oscillations is going to be affected by the noisy features of one unit. More specifically, those units which, by chance, open sooner will trigger the others generating a wave. This biases the resulting frequency toward the shortest possible for a given level of noise.

So, indeed, large cell would have slightly higher frequencies since more CaRU means more chances to have some units randomly firing sooner in the oscillation cycle determined by the single CaRU model.

*Pg15: “we consider that the inactivated states of the RyRs do not play a role in the mechanisms that produce oscillations”. This assumption needs a reference.

We are sorry for the typo. We meant to say that inactivated states do not play a role in this mechanism. This is, when we look at the full model and check the level of inactivation of the RyR we observe that they do not play a role in the oscillations. This is the reason why we do not consider inactivated states in the reduced single CaRU model used to explain oscillations.

In order to avoid any confusion to the reader, we have introduced the following changes:
-A supplemental figure that shows that indeed inactivation does not play a role in the oscillatory mechanism of the detailed subcellular model

-A paragraph explaining that this is the reason we do not use inactivated states as one of our approximations

-A new paragraph in the discussion addressing this issue which reads:

Oscillations appear when the SR Ca flux through the RyRs becomes large, either because of a large luminal calcium load or to sensitization of the RyR due to an increase in diastolic calcium. These conditions can be met from a lack of CSQ, under an increase in global cell calcium content, or upon phosphorylation of the RyR, for instance. Under these circumstances, there is a strong release flux from the SR that results in SR depletion. As the SR is refilled, the SR release flux again increases, resulting in periodic oscillations. Although RyR inactivation is present in the subcellular model, in our simulations release refractoriness is due mostly to SR depletion, and its recovery is dictated by the slow refilling of the SR since this time scale is slower than the RyRs recovery from inactivation. Thus, the latter modulates the conditions of oscillations but does not determine them. We cannot discard, however, that under conditions of fast SR refilling, RyR refractoriness could not become the determining factor of the oscillations. This is reminiscent of the situation observed in calcium alternans, where both a slow refilling of the SR and RyR inactivation have been deemed as possible mechanisms for the onset of that rhythm

*Pg16: “The parameters of these equations are taken from the literature where, except for those of the RyR, are well documented.” References are missing.

We have added some references to address this point.

*It would be helpful if authors could write all the equations and parameters in the appendix so that readers do not have to read multiple papers to reproduce models and results.

We agree. We have added a full Supplement Material with all the equation and the table of values.

*In my opinion, “We find, for instance” in the abstract is not appropriate. We want to know all the novel findings in the paper. Please enumerate all the key findings in the abstract.

We have changed the abstract following these suggestions.

*CSUN is located in Northridge (not in Los Angeles).

We have changed the location in the address.

Response to reviewer 2:

This manuscript is well written and in principle is a nicely designed sequence of model reduction for describing the dynamics underlying calcium instabilities in cardiac myocytes. However, I have considerable concern that the novelty of the findings are not sufficient to warrant publication in PLoS Comp Biol.

We are glad that the referee finds the manuscript well written and designed. Below we detail the responses to the referee’s concerns:

Major comments: The authors have provided a coherent analysis of 2 models, which are both reductions of their recently published model of subcellular calcium signaling in cardiac myocytes. In general I support their efforts to condense the complexity of the complete model to its essential dynamics. Previously this has been done to great effect for understanding EAD dynamics, and several times prior (sometimes in very similar fashion) for defining the stability of the calcium resting state in cardiomyocytes (see for example Tveito et al. 2012, Nivala et al. 2012). While I support the objectives in this way, I do not see the novelty in the current study to support publication at this time. In particular, the role of reduced SR calcium buffering in promoting instability of the diastolic resting state has been fully established experimentally and clinically as the second (and most severe) form of catecholaminergic polymorphic ventricular tachycardia (CPVT2). Similarly, the role of increased whole-cell calcium load in this form of instability has been well known for several decades, and specific critical SR calcium thresholds have been measured in a number of species (see many studies by Eisner’s group). In sum, my major comment here is that, while there has been great importance in prior studies that have used reduced models for describing the essential dynamics of instability e.g EADs, the real value of those analyses was permitted because there was an important disagreement between experimental observation, and the reigning concepts of the time. In the case of EADs this was the observation that APD could be prolonged tremendously without observing the transition to instability. I do not see any additional level of insight generated by the current study, and while it

need not be groundbreaking, I think that some new biological insight is necessary to justify consideration for publication in PLoS Comp Biol.

This is an excellent point. Indeed various studies have established the crucial role of SR load and Ca buffering on the instability of Ca release. The novel aspect of our work is that we establish that Ca buffering and RyR kinetics can interact to induce Ca oscillations. These Ca oscillations have not been explored in this specific context and may be crucial to relate CSQ mutations and cardiac arrhythmias. Thus, while instabilities have been considered in previous works, this is the first study to specifically investigate the role of Ca cycling oscillations. Furthermore, our analysis reveals that the oscillations we observe are robust and are therefore likely to be observed in an experimental setting. In the revised manuscript we have included a paragraph in the discussion explaining the novelty of Ca oscillations in the context of the cardiac Ca signaling system.

The fact that we unveil this mechanism behind the appearance of oscillations can help to explain the complicate relation between intracellular Calcium dynamics and dangerous AP repolarizations. There are different ways an abnormal calcium transient may lead to changes in AP which favor the appearance of arrhythmias. It has been quite often associated with reactivation of L-type Ca currents during repolarization. However, we have found a possible different mechanism where the appearance of an underlying oscillatory behavior when the cell loads for a given level of buffering can change the AP behavior due to its effects in L-type Ca currents and the sodium-calcium exchanger. In this sense, we establish a relation between two types of results. On the one hand there are results that describes the role of reduced SR calcium buffering in promoting instability of the diastolic resting state, but there is also literature showing that mutation in CSQ cause abnormal rhythm that are directly linked to sudden cardiac death (Viatchenko, et al 2004, Terentyev et al 2006). We have shown now that this oscillatory substrate is very robust and can have important effects under very different scenarios of RyR and buffering behavior.

We think we should have paid more attention to the implications of our work in the previous manuscript. We did put a lot of effort in showing that our mechanism was robust without clarifying why it was so important that it is so. We have introduced changes in the abstract to set the proper balance between justification of the robustness and its relevance. We have also added the following paragraph in the discussion.

The instability of the diastolic resting state is thus well-known and it plays an important role in the initiation of various cardiac arrhythmias. However, the precise mechanistic relationship between Ca and dangerous AP repolarization is still not well understood. One possibility that has been suggested in previous studies is that Ca cycling induces voltage deflections during the AP which can induce a substrate for wave break and reentry. These voltage deflections are referred to as early-after-depolarizations (EADs), which can be caused by a variety of mechanisms, such as reactivation of the L-type Ca current during AP repolarization. Our results in this paper suggest an alternative possibility that EADs may be caused by Ca oscillations that are regulated by Ca buffers such as CSQ. In this picture, oscillations of the RyR-CSQ can perturb the AP via voltage sensitive currents such as the L-type Ca current and sodium-calcium exchange. This suggests that crucial properties of EADs such as their amplitude and oscillation frequency may be determined by the Ca cycling system. This picture is consistent with several studies in the literature showing that mutations in CSQ cause abnormal Ca cycling rhythms that are directly linked to sudden cardiac death. Our study raises the possibility that Ca cycling oscillations may be a crucial component to understand these arrhythmias. This viewpoint has not been explored in the literature and may be crucial to future therapeutic approaches to control or eliminate the presence of EADs.

We hope that these changes explain better the novelty of our analysis and its relevance.

Specific comments:

1. Was there some matter of simplicity that made it easier to base most of the nullcline analyses around the assumption of rapid gating kinetics i.e. dO_Ryr/dt = 0, rather than the assumption of rapid diffusion as is assumed to assess model robustness? I ask, because this assumption (quasi-equilibrium gating) is similar to that used to construct an Eigen value analysis for describing resting calcium stability (Tveito et al. 2012). Conversely, it has generally been assumed (for a range of model reduction exercises that remain faithful for CICR), that diffusion from the cleft is approximately an order of magnitude more rapid than the gating events themselves (see papers from Hinch in 2004).

We assume that the referee is referring to the paper Tveito et al, Mathematical Biosciences 236, 97, 2012. In that paper, the authors distinguish between a slow gating of the RyR, linked to inactivation, and a fast gate, linked to the transition from the close to the open state. For the latter, the authors state that (quote): “The fast gate is not included as a state variable in the system of equations, as it is assumed to be in quasi steady state with cytosolic calcium.” This is the same approximation we do in the main part of our manuscript. We are sorry this was not clear enough in the previous version of the manuscript. Exactly the same is valid for the work by Hinch et al, Biophys J 87, 3723, 2004, where they consider fast open close dynamics, with slower inactivation of the RyR. Quoting: “This model can be simplified further by taking advantage of the fact the mean open times of the RyR

and LCC (~1 ms) are substantially shorter than the inactivation times (~20 ms) of either the LCC or RyR. The rapid equilibrium approximation is applied once again to simplify the model to a four-state model.” So we do not see any contradiction between the approximations taken in these papers and ours. What we claim in our paper is that taking that both dyadic calcium and the opening of the RyR are in equilibrium (as assumed in those papers) would eliminate the oscillations that, nevertheless, are observed in the full subcellular simulations.

In order to address this important point in the manuscript, we have introduced a supplemental figure showing that when we do not perform this simplification (and the deterministic model of the CaRUs is reduced to three variables where inactivation is included) oscillations are still present with the same type of transition. This gives further proof of the robustness of the mechanism.

-A new paragraph in the discussion addressing the issue of time scales has been added. It reads:

Oscillations appear when the SR Ca flux through the RyRs becomes large, either because of a large luminal calcium load or to sensitization of the RyR due to an increase in diastolic calcium. These conditions can be met from a lack of CSQ, under an increase in global cell calcium content, or upon phosphorylation of the RyR, for instance. Under these circumstances, there is a strong release flux from the SR that results in SR depletion. As the SR is refilled, the SR release flux again increases, resulting in periodic oscillations. Although RyR inactivation is present in the subcellular model, in our simulations release refractoriness is due mostly to SR depletion, and its recovery is dictated by the slow refilling of the SR since this time scale is slower than the RyRs recovery from inactivation. Thus, the latter modulates the conditions of oscillations but does not determine them. We cannot discard, however, that under conditions of fast SR refilling, RyR refractoriness could not become the determining factor of the oscillations. This is reminiscent of the situation observed in calcium alternans, where both a slow refilling of the SR and RyR inactivation have been deemed as possible mechanisms for the onset of that rhythm

2. With respect to the above point. The dynamics shown in Fig 5 are somewhat surprising from an intuitive perspective. This is because the SR [Ca] reached under stable conditions (C(bar)_T = 32 uM) is so much larger than for the other conditions (even though there should be no fundamental differences in the RyR properties, or in the relationship between free and total SR [Ca]). This makes me skeptical that the assumption of rapid gating is reasonable for describing the determinants of stability in this model. Can the authors explain to me what I am missing here?

The referee is completely right. This sudden decrease in SR Ca load at the onset of oscillations is not observed neither in the full subcellular model nor in experiments. It is not due to the rapid gating approximation, though, as can be confirmed simulating Eqs. (12)-(14) without assuming the rapid gating approximation for the RyR (new Supplemental Figure 1). In the new version of the manuscript we show that this discrepancy could be due to the lack of stochasticity in the minimal model. We have included a new subsection dealing with the effect of stochasticity, inherent due to the low number of RyRs present in each RyR cluster. What we show now is that the addition of a small amount of noise makes the onset of oscillations appear earlier, with a larger period, and as a gradual transition from the values of the stable state. We have also added a paragraph in the Discussion explaining this point.

The deterministic equations for the CaRU provide the proper average behavior of the units and the core behavior of the whole system. However, the period of each unit can be affected close to the transition by small amounts of noise. Due to the fact that there are a small number of RyRs per cluster, the study of periods must include an analysis of noise in the system. It provides a better representation of what occurs in the subcellular model (and in experiments). Close to the onset of a homoclinic bifurcation giving rise to a periodic orbit, a small amount of noise makes oscillations appear for values of the parameters before the onset in the deterministic limit, and with a larger period of oscillation. In fact, in the new Fig. 16 it can be seen that, at onset of oscillations, periods of the order of the second are observed. In the new version of the manuscript we have added a new subsection on the stochastic effects and modified a paragraph on the Discussion to address these results.

Introducing noise in the CaRU provides the proper oscillation period and a more realistic behavior. We would like to mention that the analysis of the deterministic minimal model is still useful to analyze the full cell since it determines the minimum period of oscillation and the possibility of having these waves in the first place. If each of the units does not have an oscillatory nature or is very close to having one, the presence of waves is not possible
