# Supplementary material for: Buffering and total calcium levels determine the presence of oscillatory regimes in cardiac cells
Source: PLoS Comput Biol. 2020 Sep 24;16(9):e1007728. doi: 10.1371/journal.pcbi.1007728 (PMC7537911; doi:10.1371/journal.pcbi.1007728)
Supplement: Attachment — Submitted filename: Response2ndReviewer.pdf [file pcbi.1007728.s005.pdf]

Dear Andrew G Edwards

We thank you for your comments. We completely agree with your statement. We just wanted to indicate that our manuscript opens us to new ideas and future work regarding EADs. We now completely clarify that a new model is needed to address this issue. The paragraph now reads:

These voltage deflections are referred to as early-after-depolarizations (EADs), which can be caused by a variety of mechanisms, such as reactivation of the L-type Ca current during AP repolarization. An alternative possibility is that EADs may be caused by Ca oscillations that are regulated by Ca buffers such as CSQ. In this picture, calcium oscillations can perturb the AP via voltage sensitive currents such as the L-type Ca current and sodium-calcium exchange. This idea is consistent with several studies in the literature showing that mutations in CSQ cause abnormal Ca cycling rhythms that are directly linked to sudden cardiac death. Thus, a natural extension of our work would be to study the effect of calcium oscillations on the AP, for which we should extend our model to include detailed, and coupled, calcium and membrane dynamics.

Sincerely.
The authors.
